# Progress in Neurology

**Published:** 1901-07-06

**Authors:** 


### PROGRESS IN NEUROLOGY.

**Paralysis Agitans.**—In the course of a paper on this disease, Williamson[1] says that the only drugs from which he has found any benefit are hyoscine hydrobromate, duboisine sulphate, and hyoscyamine.

The dose of hyoscine should not at first be more than 1-150 grain. This may be increased to 1-100 grain. If no toxic symptoms appear, such as marked dryness of the throat and dilated pupils, the dose may be cautiously increased. In some cases of paralysis agitans it may be increased little by little to 1-50 grain, but such large doses should only be given when the patient can be carefully watched. It is important to always use the same preparation of hyoscine, *e.g.* Merck's, as the strengths of different preparations vary somewhat. It is best to give it well diluted in choloroform water. It is found that hyoscine in these doses either diminishes or arrests the tremor, and checks the troublesome restlessness and desire to change position, also the unpleasant sensation of heat and flushing of the face. Sleeplessness is often a troublesome symptom in paralysis agitans. A dose of hyoscine is often of service then in diminishing the tremor and restlessness so that the patient can fall asleep. Similarly the patient may be unusually wakeful in the early morning. For this, a dose of sulphonal may be given immediately before going to bed, or hyoscine given on waking. In general it will be found that the best results of hyoscine will be obtained by thus giving it in the early morning and in the evening.

Duboisine sulphate has an action similar to that of hyoscine—in some cases it is found to be more, in others less, useful than hyoscine. Usually not more than 1-64 grain can be given without producing toxic symptoms.

Hyoscyamine sulphate does not produce dryness of the throat so readily as hyoscine; it diminishes the tremor and restlessness, though not so decidedly as hyoscine.

Though it is not possible to cure or even arrest the disease, yet these palliative measures—life in the open air as much as possible, with carriage drives, and the administration of hyoscine—render the patient's condition much more comfortable.

**Meningitis.**—Spiller[2] reports a case where symptoms of meningitis existed for six days, and yet lesions sufficient to explain these symptoms were not found at the necropsy. There was an acute onset with fever of 102°, diarrhœa, and vomiting. The symptoms of meningitis were photophobia, rigidity of the abdominal muscles, slight retraction of the head, stiffness of the extremities, erythema of the right leg, and a terminal coma. The diagnosis of cerebro-spinal meningitis was made. At the post-mortem examination no distinct evidences of inflammation of the cerebral and spinal meninges could be detected with the naked eye. A microscopical examination of the central nervous system showed little evidence of inflammation. The nerve cells throughout the brain and spinal cord were greatly altered. The cell-body was swollen and rounded, many of the dendritic processes had disappeared, the chromophilic elements were not to be seen except surrounding the nucleus which had moved to the periphery of the cell-body. The small vessels of the pia and anterior and posterior roots were much distended with blood. Numerous small bacilli were found within the nervous tissue. Spiller had previously seen the same general alteration of the nerve-cell bodies of the central nervous system in a case of internal hæmorrhagic pachymeningitis. A similar condition has been observed by Hirsch in a case of amaurotic family idiocy, and he came to the conclusion that amaurotic family idiocy is therefore an acquired disease and is produced by some poison, possibly by a toxic condition of the mother's milk. The symptoms of cerebro-spinal meningitis sometimes occur in typhoid fever and other infectious diseases without alteration of the meninges, and Spiller says that it is not impossible that in some of these cases a similar alteration of the nerve-cell bodies occurs.

Thus, not only may even purulent meningitis exist without any symptoms, but pronounced symptoms of meningitis be present with very slight alteration of the meninges.

**Cerebellar Hæmorrhage.**—An interesting case of cerebellar hæmorrhage has recently been recorded by Thyne.[3] A man, aged 20, was suddenly seized with vomiting and frontal headache. Within six hours of the onset the head was found markedly retracted. There was no loss of consciousness, no motor paralysis, no twitchings or convulsions. The tendon jerks were increased. Kernig's sign was well marked. There was no ocular deviation. The temperature was normal or subnormal, and the pulse varied from 60 to 160, but was regular in force and rhythm. The patient remained in this condition for twelve days, except that on the day preceding death he lost power in both legs. At the post-mortem examination a hæmorrhage in the left lateral lobe of the cerebellum was found, which had burst into the fourth ventricle. The case is of much interest from the fact that well-marked cervical opisthotonus and Kernig's sign were due to cerebellar hæmorrhage—meningitis, with which they are usually associated, being absent.

[1] Med. Chron., Feb. 1901. [2] Jour. of Nerv. and Ment. Dis., March, 1901. [3] Lancet, Feb. 9, 1901.

